# Building Blocks for Deep Phenotyping in Infancy: A Use Case Comparing Spontaneous Neuromotor Functions in Prader-Willi Syndrome and Cerebral Palsy

**DOI:** 10.3390/jcm12030784

**Published:** 2023-01-18

**Authors:** Dajie Marschik-Zhang, Jun Wang, Xiushu Shen, Xiaoyun Zhu, Herong Gao, Hong Yang, Peter B. Marschik

**Affiliations:** 1 Child and Adolescent Psychiatry and Psychotherapy, University Medical Center Göttingen, 37075 Göttingen, Germany; 2 Leibniz Science Campus Primate Cognition, 37077 Göttingen, Germany; 3 iDN—Interdisciplinary Developmental Neuroscience, Division of Phoniatrics, Medical University of Graz, 8036 Graz, Austria; 4 Department of Rehabilitation, Children’s Hospital, Fudan University, Shanghai 201102, China; 5 Center of Neurodevelopmental Disorders (KIND), Centre for Psychiatry Research, Department of Women’s and Children’s Health, Child and Adolescent Psychiatry, Region Stockholm, Karolinska Institutet & Stockholm Health Care Services, 17176 Stockholm, Sweden

**Keywords:** deep phenotyping, cross-condition comparison, infant, neuromotor function, motor development, Prechtl general movements assessment (GMA), motor optimality score-revised (MOS-R), Prader-Willi syndrome (PWS), cerebral palsy (CP)

## Abstract

With the increasing worldwide application of the Prechtl general movements assessment (GMA) beyond its original field of the early prediction of cerebral palsy (CP), substantial knowledge has been gained on early neuromotor repertoires across a broad spectrum of diagnostic groups. Here, we aimed to profile the neuromotor functions of infants with Prader-Willi syndrome (PWS) and to compare them with two other matched groups. One group included infants with CP; the other included patients who were treated at the same clinic and turned out to have inconspicuous developmental outcomes (IOs). The detailed GMA, i.e., the motor optimality score-revised (MOS-R), was used to prospectively assess the infants’ (*N* = 54) movements. We underwent cross-condition comparisons to characterise both within-group similarities and variations and between-group distinctions and overlaps in infants’ neuromotor functions. Although infants in both the PWS and the CP groups scored similarly low on MOS-R, their motor patterns were different. Frog-leg and mantis-hand postures were frequently seen in the PWS group. However, a PWS-specific general movements pattern was not observed. We highlight that pursuing in-depth knowledge within and beyond the motor domain in different groups has the potential to better understand different conditions, improve accurate diagnosis and individualised therapy, and contribute to deep phenotyping for precision medicine.

## 1. Introduction

Over the past few decades, with the increasing worldwide application of the Prechtl general movements assessment (GMA) beyond its original field of early prediction of cerebral palsy, substantial knowledge has been gained on early neuromotor repertoires across a broad spectrum of neurodevelopmental disorders, congenital diseases, and genetic syndromes [1,2,3,4,5,6]. We have learned that age-inadequate, monotonous, and abnormal general movements signal deviances in the developing young nervous system. Especially the absence of fidgety movements (FMs, an age-specific spontaneous motor pattern of general movements; [1]) from 3 to 5 months after term flags a high risk for adverse neurodevelopmental outcomes [7].

As known, different parts of the human brain become specialised for different functions during development. The newborn’s brain starts with highly interconnected cortical regions and shows a considerable degree of malleability. The modularisations of the functional structures are yet to unfold [8]. The size of some functional areas in the young brain can thus increase or decrease depending on the activities and experience of an individual [9]. The development of neuromotor functions, for instance, continuously interacts with the neurofunctions in other developmental domains (e.g., sensory processing, executive control, language processing, social cognition). These dynamics progressively shape the individual’s developing brain and cascade further development throughout the first months and years of life [10].

Not surprisingly, an infant’s early motor repertoire was found to be associated with the individual’s later development in executive control, language, and cognitive functions beyond the motor domain [11,12,13,14]. Compared with behaviours in other developmental domains (e.g., vocalisations), young infants’ spontaneous movements are constantly observable during their active wakefulness [15,16], providing a readily accessible source for evaluating the integrity of the developing brain. Assessing infants’ motor behaviours with GMA, for example, may hence give valuable cues not only for individuals’ concurrent brain development but also for predicting their long-term neurofunctional outcomes.

Applying the detailed general movements assessment, the motor optimality score (MOS, [17]), a previous case report documented the motor development of a male infant with Prader-Willi syndrome (PWS) during his first 6 months of life, adding to the literature of using GMA to describe the spontaneous motor repertoire of yet another diagnostic group [18]. PWS is a rare genetic disorder with a birth incidence of 1/8000 to 1/30,000. It is caused by the absence of expression of the paternally derived chromosome 15 at locus q11–q13 [19]. The disorder is characterised by infantile hypotonia, feeding difficulty, and lethargy, followed by a wide range of physical (e.g., hypogonadism, short stature, obesity), neuropsychiatric (e.g., excessive eating, repetitive and ritualistic behaviours, obsessiveness, temper outbursts, brief mood swings, self-injury), and intellectual impairments emerging from early childhood onwards [20]. While these phenotypical characteristics of PWS have been mentioned, structured descriptions of the neuromotor functions of this disorder in early infancy are scarce. According to the case report by Pansy and colleagues [18], the infant’s motor repertoire was characterised as monotonous, severely reduced in variety, and age inadequate. While in typically developing infants, the age-specific fidgety movements are commonly present from 9 to 20 weeks of post-term age [7,16], in this infant with PWS, fidgety movements did not emerge until 17 weeks and were still observable at 27 weeks post term.

The current study aimed to complement the previous case study with a larger sample to better understand and profile the neuromotor functions of infants with PWS. We used the manualised detailed GMA, i.e., the motor optimality score-revised (MOS-R) [18], to analyse infants’ spontaneous motor repertoires. We implemented cross-condition comparisons to characterise both within-group variations and between-group differences and overlaps in infants’ neuromotor functions. We intend to highlight that pursuing in-depth knowledge within and beyond the motor domain of different genetic and neurodevelopmental disorders has the potential to support accurate diagnosis and tailored intervention and to contribute to deep phenotyping for precision medicine.

## 2. Materials and Methods

### 2.1. Participants

Data acquisition was conducted at the Rehabilitation Department of the Children’s Hospital of Fudan University in Shanghai, China. The Rehabilitation Department serves between 10,000 and 20,000 visits of young children annually. In 2014, a standard infant-care programme was established, with GMA embedded in routine clinical care for screening, diagnostics, and surveillance. Within this programme, more than 2000 patients from 0 to 5 months of age with special needs and perinatal complications are treated yearly and routinely evaluated with GMA at the Rehabilitation Department. From 2014 to the end of 2021, 27 children with PWS were registered and their data can be retrieved from the archive of the Rehabilitation Department. Inclusion criteria of the current study for infants with PWS included (a) the family of the infant signed the informed written consent for study participation; (b) genetic testing confirmed the diagnosis of PWS in the infant; (c) a brain MRI of the infant did not reveal severe brain injury and the patient did not receive a diagnosis of cerebral palsy (please also see below for characteristics of the comparison groups); and (d) the infant was prospectively evaluated with GMA between 9 and 20 weeks of post-term age. Of the retrievable cases, 18 met the inclusion criteria and were enrolled in the study. Deletion of paternal inherited genes was confirmed by genetic testing in 10 infants, and maternal uniparental disomy (mUPD) was verified in the other 8 infants. Diagnostic age ranged from 2 weeks to 8 months after birth (median = 10 weeks, IQR 5–19 weeks). The majority of the patients (13/18) came from the same hospital (e.g., neonatal ward) to the Rehabilitation Department. The other 5 patients were interhospital referrals. Of the 18 infants, 7 were evaluated with GMA before the genetic and clinical diagnosis of PWS was made.

From the same archive, 2 comparison groups with 18 infants each were likewise retrieved. Infants from both groups were patients who experienced various perinatal complications and were treated at the Rehabilitation Department (please see the Supplementary Materials for anamneses of the patients). One group of children were later diagnosed with cerebral palsy (CP). The other group included children who turned out to have inconspicuous developmental outcomes (IOs; outcomes were confirmed through both clinical evaluations and standardised assessments adapted for the Chinese population, including the Griffith developmental mental scales, the Peabody developmental motor scales-2, the symbolic play test, and the Reynell developmental language scales). Both groups were matched to the PWS group by gestational age at birth and corrected age at GMA (Table 1). Gender and ethnicity were not matched, because neither has been indicated as being related to general movements [16]. For both comparison groups, GMA was routinely implemented at the Rehabilitation Department at a time when developmental outcomes of the infants were unknown.

The final sample with three groups (PWS, CP, and IOs) enrolled 54 infants in total. All infants received individualised outpatient treatment at the Rehabilitation Department. The infants were followed up with or received further therapies either at the Rehabilitation Department or at the patients’ local medical care institutions up to 2 years of age and, if applicable, beyond. Parents of all enrolled infants gave their written informed consent to participate in the study. Ethical approval was attained from the Research Ethical Board of the Children’s Hospital of Fudan University, Shanghai, China.

### 2.2. Procedure

All 54 infants were prospectively video recorded according to the GMA standards [16]. Recordings lasted between 3 and 7 min (median = 4 min). Infants’ post-term ages at GMA ranged from 9 to 20 weeks (median = 12). Three GM-Trust advanced-level trained and certified raters, two physiotherapists (rater A and B), and a rehabilitation physician (rater C) independently performed the MOS-R, i.e., the detailed GMA. Raters A and B were blind of the medical history of all the infants. They were informed only of the infant’s age at GMA. Rater C, the rehabilitation physician of the infants, was aware of the background of the patients at the time of scoring. As GMA was routinely implemented during the first months of life of the infants, none of the raters knew the later developmental outcomes of the infants. Interrater reliabilities for the overall MOS ranged from 0.86 to 0.95 (Intraclass correlation coefficients, pairwise agreement), and intrarater reliabilities were higher than 0.95 for all the three raters [21]. In case of discrepancy, a consensus score was attained for further analyses, for the current study.

### 2.3. Statistics

SPSS (Version 21, Chicago, IL, USA) was used for processing the data. The total score of MOS-R and its subscale scores are ordinal data. Mann–Whitney U test was used to determine whether two independent samples had the same distribution in MOS and its subscales and whether the distributions of normal vs. abnormal movement patterns, postures, or movement characters (dependent samples, not normally distributed) were comparable. Student’s *t*-test was applied to compare the total number of observable movements between two groups or to compare interval variables between groups, such as age or birth weight. Statistical significance was assumed if *p* < 0.05.

## 3. Results

### 3.1. Spontaneous Motor Patterns of Infants with PWS

The infants with PWS generally appeared inactive and lethargic. The majority (12/18) moved only infrequently or intermittently, although they were awake. The infants presented a very limited number of spontaneous movement patterns that are commonly observable in typically developing infants at this age and are scorable with MOS-R [17]. As shown in Table 2, the MOS of the 18 infants with PWS ranged from 6 to 18 points. None scored in the optimal range of 25–28. Most infants (15/18) scored 6 or 7 points. Only one infant (aged 15 weeks) presented normal fidgety movements, with a total score of 18. Another one (aged 9 weeks) presented “fidgety-like” movements with reduced speed, with a total score of 13. Four of the patients (4/18) had already received growth-hormone therapy by the time of GMA, three of whom, like the majority of the other PWS cases, scored 6 or 7 points. The MOS of infants identified with mUPD (*n* = 8) and that of the infants with a deletion of paternal inherited genes (*n* = 10) was not significant (Mann–Whitney U = 31.5, Z = 0.71, *p* = 0.48).

On average, only about two scorable patterns (range: 0–6), including both normal and abnormal ones, could be observed in infants with PWS. None of the 18 infants presented an age-adequate movement repertoire. The majority (10/18) did not present any normal movement pattern, one-third (6/18) presented a single normal movement pattern, and only two infants each presented three normal patterns. Only two infants did not present any abnormal pattern, one of whom also did not present any normal pattern. The other 16 infants each presented one to four abnormal patterns. Movements towards midline or antigravity movements were seldomly observed. Only one infant presented normal foot-to-foot contact, two presented normal leg lift, and none presented normal hand-to-hand contact. Most expected age-specific movements (i.e., items on MOS-R) were not observed in normal forms in any of the 18 infants (e.g., swipes, kicking, excitement bursts, side-to-side head movements, hand–mouth contact, fiddling, reaching, hand–knee contact, arching, rolling to side, head anteflexion). Normal wiggling-oscillating, smiles, mouth movements, visual exploration, and hand regard were observed respectively in only one or at most two of the 18 infants. Half of the infants (9/18) hardly moved their legs (i.e., almost no leg movements) during recording. Abnormal side-to-side head movements (e.g., repetitive rotation), visual exploration (e.g., strabismus), and asymmetric finger postures were each observed in three or four infants. Other movement patterns in abnormal forms were occasionally observed in one or two infants.

**Figure 1:**
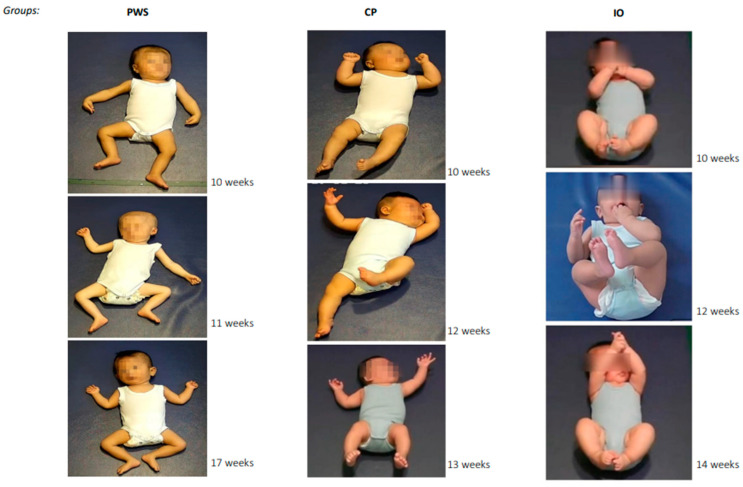
Examples of postural patterns from the Prader-Willi syndrome (PWS), cerebral palsy (CP), and inconspicuous developmental outcomes (IOs) groups, respectively. Three infants are exemplified for each group. Pictures were extracted from the original GMA videos. The infant’s post-term age at the time of GMA is provided next to each picture. The “frog-leg” posture of the lower extremities presented in all the three examples of the PWS group were observed in 13 of the 18 infants in the group. About one-third (7/18) of the infants also presented long-lasting or repetitive “mantis-hand” postures (unilateral or bilateral pronated wrist(s) with extended adducted fingers), as shown on the top example of the PWS group.

Regarding posture, none of the 18 infants presented more normal than abnormal postures. Two-thirds (12/18) lay their body and limbs flat on a surface (flat posture). Only three of the infants could hold their head in midline, and two could keep body symmetry. Asymmetric tonic neck (ATN) was never observed. Only one infant presented variable normal finger postures. The majority (14/18) showed very few finger postures. A few presented finger spreading (3/18) or predominant fisting (1/18). Extended legs (6/18) and/or arms (3/18) on the lying surface were also observed. Illustrated in Figure 1, more than two-thirds of the infants (13/18) presented a predominant “frog-leg” lower-extremity posture, with bilateral externally rotated abducted hips, flexed knees, and externally rotated ankles. More than one-third (7/18) of the infants also presented a “mantis-hand” posture, with unilateral or bilateral pronated wrist(s) with extended adducted fingers (i.e., top-left picture).

The movements of all 18 infants appeared monotonous. Five infants’ movements were also rated as jerky. Most infants (11/18) moved with a predominantly slow speed.

### 3.2. Spontaneous Motor Patterns of Infants with PWS Compared with Infants with CP and Infants with Inconspicuous Outcomes (IOs)

As mentioned before, the 54 infants in the current sample were patients treated at the Rehabilitation Department (see Supplementary Materials). At the time of GMA, the diagnoses and outcomes of the patients in the CP and IOs groups were unknown. Here, we focused on our group comparisons between PWS and CP, and, between PWS and IOs. The comparisons between CP and IOs were skipped, as this was not the target of the current study and was done in previous studies [17].

Shown in Table 2, neither the total score of MOS-R nor the scores of all its subscales were significantly different between the PWS and the CP groups. Nearly all the scores were significantly different between the PWS and the IOs groups, except for movement character. This reflects the general poor well-being of the young infants in both the PWS and the CP groups, in contrast to the same-aged infants who also experienced diverse pre-or perinatal complications but later developed inconspicuously (i.e., IOs group; please see the Supplementary Materials for anamneses of the patients).

As reported above, the infants with PWS in general moved only rarely. The number of observable movement patterns (including both normal and abnormal ones) of an infant with PWS (M = 2.44, SD = 1.62) was significantly less than that of an infant with CP (M = 3.83, SD = 1.34), t(34) = −2.81, *p* < 0.01; and still less than that of an infant in the IOs group (M = 4.72, SD = 1.60), t(34) = −4.25, *p* < 0.001. In both the PWS and the CP groups, the movement repertoire was rated as “absent” in nearly all infants (Table 2, Repertoire). The movement repertoire in the IOs group was more frequently rated as age adequate (4/18) or reduced (9/18). In both the PWS and the CP groups, more infants presented predominantly abnormal movement patterns, while all infants in the IOs group presented more normal than abnormal patterns (Table 2, Other Movements).

Despite the similarly poor scores on MOS-R and its subscales of the PWS and the CP groups, their spontaneous motor patterns were different in several ways. Table 3 presents the most frequently observed movement and postural patterns and movement characters in the three groups of infants.

As can be seen, except for “almost no leg movements” (9/18), no movement pattern (neither a normal nor an abnormal one) was observed in more than four infants in the PWS group. In contrast, a variety of normal movement patterns and very few abnormal ones were observed in the IOs group. Abnormal mouth and tongue movements and side-to-side head movements were most frequently observed in the CP compared with the other groups.

Also shown in Table 3 (Postural Pattern), although the majority of the infants in the IOs group kept their head centred and body symmetric, presented variable finger postures, and otherwise rarely showed any abnormal postural patterns, the majority of the infants in the PWS and the CP groups presented more abnormal than normal postures (see also Table 2, Posture). In both groups, most infants were unable to keep their head centred or their body symmetric, and most presented abnormal finger postures. While 13 infants in the PWS group presented a flat body posture, none of the infants in the CP or in the IOs group did. In contrast, numerous infants in the CP group presented the hyperextension of neck and/or trunk and predominant fisting, which were rarely observed in the PWS or the IOs group. Although extended arms and legs were observed in the PWS and the CP groups, video recordings revealed that the infants in the PWS group lay their arms and legs feebly *on* the surface, whereas the infants in the CP group extended the arms and legs mostly stiff and tensely *above* the surface or in the air (Figure 1).

Presented in Table 3 (Movement Character), none of the infants in the PWS or the CP groups moved smoothly and fluently. Nearly all the infants in the two groups moved monotonously. In the CP group, several infants’ movements were additionally rated as jerky, stiff, or tremulous. In the PWS group, predominantly slow speed was recurrently seen. One-third of the infants in the IOs group moved smoothly and fluently. The others’ movements were also rated as monotonous and/or jerky.

## 4. Discussion

Over the past few decades, not only have technological possibilities for the automated classification of general movements evolved and artificial intelligence and machine learning been increasingly applied [6,22,23,24,25,26], but also the clinical knowledge on general movement patterns have become more differentiated and fine-grained [17,27]. Especially with the MOS-R, the extension of the classic GMA, as assessment tool, a variety of studies have described the spontaneous motor patterns of infants with divergent medical backgrounds [27]. As a unique tool for evaluating age-specific movement repertoires and their quality, the MOS-R enables clinicians and researchers to profile and compare the neuromotor functions of infants from different diagnostic groups. Such knowledge is essential for gaining an in-depth understanding of the medical conditions and their different phenotypes.

In countries with advanced medical care systems, PWS, as a genetic condition, can already be diagnosed within the first weeks of life [28]. This is yet not necessarily the case in many middle- and low-resource countries and regions. In these settings, a reliable and efficient diagnostic tool such as GMA is invaluable [29]. By carefully assessing infants’ spontaneous movements, suspicions of individual developmental deviances can be denied or confirmed during the first months of life, increasing the chance of timely identifying and treating conditions with adverse prognoses.

Sampling a reasonable number of cases of a rare disease such as PWS within a reasonable time might be possible only in a multicentred study or in a high-population country. The latter was the case in the current study. As a complement to the previous case study, we recruited 18 infants with PWS and compared them to two other matched groups to develop an in-depth and comprehensive profile of the early neuromotor repertoire of PWS. We confirmed a range of findings reported in the previous case study [18], as well as added knowledge on the neuromotor functions of PWS that could not be gained from a single case (please see below). We chose CP as a comparison group because research with GMA has accumulated consolidated knowledge of this group, which we believe to be a reliable reference for cross-condition comparisons of infants’ spontaneous movements.

In our study, detailed GMA was implemented during clinical routines and at a time when infants from all three groups were treated at the Rehabilitation Department and when their long-term outcomes were unknown. In line with the literature, the MOS-R could clearly distinguish inconspicuous versus adverse developments at this early stage. Infants in the IOs group all had a total MOS higher than 20 points, while none in the PWS or the CP group did. Only one infant (aged 15 weeks post term) with PWS presented normal fidgety movements. As reported in the previous case study [18], the infant with PWS did not present fidgety movements until 17 weeks. In our sample, none of the five infants aged 17 weeks or older presented fidgety movements. As we do not have further videos from the infants, we cannot exclude the possibility that some or all 18 infants in the PWS group presented fidgety movements at an older age. Nonetheless, according to our observations, most infants in the PWS group did not present fidgety movements at an age when typically developing infants do. And the only infant with normal fidgety movements, like the infant in the previous case report, performed otherwise poorly and presented an age-inadequate movement repertoire. Future observations shall clarify whether there is a general delay in fidgety movements in infants with PWS or, more likely, a deviation, in which fidgety movements might never present in some. Further, it would be interesting to find out whether an absence of (instead of a delay in) fidgety movements in PWS, if observed, is associated with severer neurodevelopmental deficits later in life.

In the current study, we refrained from examining the predictivity of MOS-R on developmental outcomes for two reasons, which we also see as limitations of this work. First, infants in this cohort were diagnosed with PWS at different ages, ranging from 2 weeks to 8 months after birth. After clinical diagnosis, some infants immediately received growth-hormone therapy, while some did not. The main reason was that the treatment is not covered by health insurance and has to be paid by the patient’s family. The timing and intensity of this and other treatments were also often dependent not only on the severity of the infant’s condition but also on the financial capacity of the family. The different therapies could considerably influence the developmental trajectories and outcomes of the infants. Second, after receiving the initial treatment at the Rehabilitation Department in Shanghai, some patients moved back to their home towns. The developmental outcomes of the infants were then measured by different institutions with different tools at different times, making comparisons barely feasible. Another limitation of the study is that we do not have detailed information on the hypothalamic dysfunctions of the patients, which is associated with the clinical symptoms of PWS [19]. Thus, unfortunately, we cannot analyse the potential relationship between general movements of the infants and the hypothalamic disorders. Nonetheless, the clinical and genetic diagnoses of PWS were confirmed in all infants in the group and analyses of their general movements can be reliably carried out.

In line with the literature, the clinical records of most infants in the current PWS group documented feeding difficulties and hypotonia (see Supplementary Materials). This was also reflected in our observation of their motor patterns. Similar to what was reported in the previous case study, the majority of infants in the PWS group appeared lethargic, moved rarely, and accordingly presented a severely reduced movement repertoire. Their posture in most cases was flat and floppy, echoed by their monotonous and slow-speed movement character. Not having been itemised on MOS-R, the frog-leg posture of the lower extremities, which indicates a generalised reduction in muscle tone [30], and the mantis-hand posture were frequently observed in the current PWS group (Figure 1).

Although infants in both the PWS and the CP groups had similarly poor scores on MOS-R, their motor pattens were clearly different. Compared with the PWS group, infants with CP presented a greater number of movement patterns. In the CP group, the frequently observed movement patterns (e.g., repetitive tongue protrusion, side-to-side head movements), postures (e.g., hyperextension of neck and trunk, predominant fisting, extended arms), and movement characters indicated stiffness and tension in most infants.

Although all the infants with PWS scored well below the optimal range of 25–28 points [17], reflecting their general compromised well-being, not all infants in the PWS group appeared lethargic or inactive. The absence of leg movement and the flat posture were common, but neither was seen in all infants of the group. Moreover, inactiveness, reduced motor repertoire, and floppy posture were also frequently observed in infants with infantile hypotonia in numerous diagnostic groups (e.g., Down syndrome, Rett syndrome, spinal muscular atrophy, and William syndrome [31]). In other words, a PWS-specific general movement pattern cannot be claimed and may not exist. Rather, while some core movement features were presented in most infants with PWS, individual variations were as well observed. These within-syndrome heterogeneity and across-condition similarities continuously pose challenges for specific and timely identifications.

Still, by scrutinising cross-condition overlaps and distinctions, we are likely to get closer to the specific fingerprints of distinctive disorders and their subphenotypes [32]. Deep phenotyping requires input not only at the structural and molecular levels but also at the functional level; each assembles different pieces towards solving an intricate puzzle. As genetic testing may identify alterations in genes, it cannot determine their phenotypical realisations. Functional specifications and modularisations of the brain are partly predefined and partly determined by individual experiences and activities from the first day onwards [33]. The constant interactions of different parts of the brain enable and also constrain basic and complex neurofunctions of an individual. With the current study, we exemplified another use case, Prader-Willi syndrome, of applying the detailed general movements assessment and cross-condition comparisons to systematically analyse and profile infant neuromotor functions, highlighting both groupwise characteristics and within-group variabilities.

Such efforts of pooling various pieces of evidence both within and beyond the motor domain are particularly required for understanding conditions which cannot be identified through genetic screening and are known for their heterogeneous phenotypic manifestations, such as autism spectrum disorder [34]. These complex disorders are often identified rather late, at preschool age or older. Insights gained from the motor domain, as from the current study, in addition to that from the other domains, will add important building blocks to deep-phenotyping different genetic and neurodevelopmental conditions to inform and improve diagnoses and individualised therapies for precision medicine.

## Figures and Tables

**Table 1:** Demographical and clinical characteristics of infants with Prader-Willi syndrome (PWS), cerebral palsy (CP), or inconspicuous developmental outcomes (IOs). Each group had 18 cases.

Variable	Mean ± SD; N (%); or Median and Interquartile (Range)			*p*-Values ^a^	
	PWS	CP	IOs	PWS vs. CP	PWS vs. IOs
Maternal Age	34.2 y ± 4.4	29.9 y ± 3.8	33.9 y ± 3.2	0.01	0.87
Paternal Age	31.5 y ± 5.2	28.2 y ± 3.3	33.1 y ± 2.9	0.05	0.33
Male Gender	14 (77.8%)	11 (61.1%)	13 (72.2%)	n.a.	n.a.
Parity	Median = 1; P25 = 1; P75 = 2	Median = 1; P25 = 1; P75 = 2	Median = 1; P25 = 1; P75 = 2	n.a.	n.a.
	(1–3)	(1–2)	(1–3)		
Preterm Birth	3 (16.7%)	3 (16.7%)	3 (16.7%)	n.a.	n.a.
Caesarean Section	11 (61.1%)	7 (38.9%)	4 (22.2%)	n.a.	n.a.
Birth Weight	2698.7 ± 448.1	3110.8 ± 606.8	3061.39 ± 765.6	0.03	0.12

*Key*: y, years; n.a., not applicable; **^a^** Student’s *t*-test.

**Table 2:** The motor optimality score (MOS) and its subcategories in infants diagnosed with PWS, those with cerebral palsy (CP), and in infants with later inconspicuous developmental outcomes (IOs). Each group had 18 cases.

	PWS	CP	IOs	*p*-Values ^a^
**Recording age**				
9–12 weeks	7	7	7	
13–16 weeks	6	6	6	n.a.
17–20 weeks	5	5	5	
**MOS**	Median = 6	Median = 7	Median = 24	PWS vs. CP:
	P25 = 6, P75 = 7	P25 = 6, P75 = 9	P25 = 24, P75 = 26	*p* > 0.05
	Range: 6–18	Range: 6–9	Range: 21–28	PWS vs. N: *p* < 0.01
**Fidgety movements**				
Normal	1	0	18	PWS vs. CP:
Abnormal	1	0	0	*p* > 0.05
Absent	16	18	0	PWS vs. N: *p* < 0.01
**Other Movements**				
N > A	2	5	18	PWS vs. CP:
N = A	2	6	0	*p* > 0.05
N < A	14	7	0	PWS vs. N: *p* < 0.01
**Age-adequate Repertoire**				
Present	0	0	4	PWS vs. CP:
Reduced	1	0	9	*p* > 0.05
Absent	17	18	5	PWS vs. N: *p* < 0.01
**Posture**				
N > A	0	0	15	PWS vs. CP:
N = A	2	0	1	*p* > 0.05
N < A	16	18	2	PWS vs. N: *p* < 0.01
**Movement character**				
Smooth, fluent	0	0	6	
Abnormal, not CS	18	18	12	PWS vs. N:
CS	0	0	0	*p* > 0.05

*Key*: N > A, more normal than atypical patterns; N = A, an equal number of normal and atypical patterns; N < A, fewer normal than atypical patterns; CS, cramped-synchronised movement character, i.e., spontaneous general movements appear stiff; limb and trunk muscles almost simultaneously contract and then almost simultaneously relax [16]; n.a., not applicable (matching criterion). ^a^ Mann–Whitney U test.

**Table 3:** Frequently observed normal (N) vs. abnormal (A) Movement patterns, postural patterns, and movement characters in the three groups of infants (with inconspicuous developmental outcomes, IOs; Prader-Willi syndrome, PWS; or cerebral palsy, CP). Each group had 18 cases. An MOS-R item is listed only if it was observed in at least five infants in one of the three groups. Numbers indicate the observed frequency. The cell is left empty if the pattern was not observed in the respective group. The highest frequency among the three groups for the respective comparison is formatted bold and italic.

Subscale		Group	Item on MOS-R									
**Movement Pattern**			Wiggling-Oscillating	Smiles	Mouth Movements	Tongue Movements ^a^	Side-to-Side Movements of the Head	Hand-to-Mouth Contact	Foot-to-foot Contact	Leg Lift	Visual Exploration	Almost No Leg Movements ^a^
	N	IOs	***6***	***5***	***12***		***13***	***5***	***9***	***12***	***11***	
		PWS	1	1	3		1		1	2	2	
		CP	2	4	8		3	2		2	7	
	A	IOs			1	2						
		PWS	1	3		1	4	2	1	1	***3***	***9***
		CP	***3***		***9***	***12***	***6***					
**Postural Pattern**			Head centred	Body symmetry	Asymmetric tonic neck	Flat posture ^a^	Variability of finger postures	Predominant fisting ^a^	Hyperextension of neck ^a^	Hyperextension of trunk ^a^	Extended arms ^a^	Extended legs ^a^
	N	IOs	***13***	***12***	***18***		***16***					
		PWS	3	2	***18***		1					
		CP	6		12							
	A	IOs	5	6								
		PWS	***15***	16		***13***	17	1	2		3	***5***
		CP	12	***18***	6	2	***18***	***5***	***14***	***8***	***7***	3
**Movement** **Character**			Smooth and fluent	Monotonous ^a^	Jerky ^a^	Stiff ^a^	Predominantly slow ^a^					
	-	IOs	***6***	7	***7***							
		PWS		***18***	5	1	***11***					
		CP		17	6	***5***						

^a^ Item targets abnormal patterns only.

## Data Availability

The datasets generated and analysed during the current study are available from the corresponding author on reasonable request. The video recordings of the individual participants are confidential and not available for public access.

## Supplementary Materials

The following supporting information can be downloaded at: https://www.mdpi.com/article/10.3390/jcm12030784/s1, Table S1: Perinatal risk factors in infants with Prader-Willi syndrome (PWS), those with cerebral palsy (CP), and infants with inconspicuous developmental outcomes (IOs).

## References

[1] Prechtl H.F.R., Einspieler C., Cioni G., Bos A.F., Ferrari F., Sontheimer D. (1997). An early marker for neurological deficits after perinatal brain lesions. Lancet.

[2] Marschik P.B., Soloveichick M., Windpassinger C., Einspieler C. (2015). General movements in genetic disorders: A first look into Cornelia de Lange syndrome. Dev. Neurorehabil..

[3] Herrero D., Einspieler C., Panvequio Aizawa C.Y., Mutlu A., Yang H., Nogolova A., Pansy J., Nielsen-Saines K., Marschik P.B. (2017). The motor repertoire in 3- to 5-month old infants with Down syndrome. Res. Dev. Disabil..

[4] Einspieler C., Utsch F., Brasil P., Panvequio Aizawa C.Y., Peyton C., Hydee Hasue R., Francoso Genovesi F., Damasceno L., Moreira M.E., Adachi K. (2019). Association of infants exposed to prenatal zika virus infection with their clinical, neurologic, and developmental status evaluated via the general movement assessment tool. JAMA Netw. Open.

[5] Palchik A.B., Einspieler C., Evstafeyeva I.V., Talisa V.B., Marschik P.B. (2013). Intra-uterine exposure to maternal opiate abuse and HIV: The impact on the developing nervous system. Early Hum. Dev..

[6] Silva N., Zhang D., Kulvicius T., Gail A., Barreiros C., Lindstaedt S., Kraft M., Bölte S., Poustka L., Nielsen-Saines K. (2021). The future of general movement assessment: The role of computer vision and machine learning-a scoping review. Res. Dev. Disabil..

[7] Einspieler C., Peharz R., Marschik P.B. (2016). Fidgety movements-tiny in appearance, but huge in impact. J. Pediatr..

[8] Westermann G., Mareschal D., Johnson M.H., Sirois S., Spratling M.W., Thomas M.S.C. (2007). Neuroconstructivism. Dev. Sci..

[9] Sur M., Rubenstein J.L.R. (2005). Patterning and plasticity of the cerebral cortex. Science.

[10] D’Souza D., Karmiloff-Smith A. (2011). When modularization fails to occur: A developmental perspective. Cogn. Neuropsychol..

[11] Einspieler C., Bos A.F., Libertus M.E., Marschik P.B. (2016). The general movement assessment helps us to identify preterm infants at risk for cognitive dysfunction. Front. Psychol..

[12] Hitzert M.M., Roze E., Van Braeckel K.N., Bos A.F. (2014). Motor development in 3-month-old healthy term-born infants is associated with cognitive and behavioural outcomes at early school age. Dev. Med. Child Neurol..

[13] Salavati S., Bos A.F., Doyle L.W., Anderson P.J., Spittle A.J. (2021). Very preterm early motor repertoire and neurodevelopmental outcomes at 8 years. Pediatrics.

[14] Salavati S., Einspieler C., Vagelli G., Zhang D., Pansy J., Burgerhof J.G.M., Marschik P.B., Bos A.F. (2017). The association between the early motor repertoire and language development in term children born after normal pregnancy. Early Hum. Dev..

[15] Einspieler C., Marschik P.B., Prechtl H.F.R. (2008). Human motor behavior. Z. Für. Psychol. J. Psychol..

[16] Einspieler C., Prechtl H., Bos A., Ferrari F., Cioni G. (2004). Prechtl’s Method on the Qualitative Assessment of General Movements in Preterm, Term and Young Infants.

[17] Einspieler C., Bos A.F., Krieber-Tomantschger M., Alvarado E., Barbosa V.M., Bertoncelli N., Burger M., Chorna O., Del Secco S., DeRegnier R.A. (2019). Cerebral palsy: Early markers of clinical phenotype and functional outcome. J. Clin. Med..

[18] Pansy J., Barones C., Urlesberger B., Pokorny F.B., Bartl-Pokorny K.D., Verheyen S., Marschik P.B., Einspieler C. (2019). Early motor and pre-linguistic verbal development in Prader-Willi syndrome-A case report. Res. Dev. Disabil..

[19] Cassidy S.B., Schwartz S., Miller J.L., Driscoll D.J. (2012). Prader-Willi syndrome. Genet. Med..

[20] Whittington J., Holland A. (2010). Neurobehavioral phenotype in Prader-Willi syndrome. Am. J. Med. Genet. C Semin. Med. Genet..

[21] Wang J., Shen X., Yang H., Shi W., Zhu X., Gao H., Yin H., Meng F., Wu Y. (2022). Inter- and intra-observer reliability of the "Assessment of Motor Repertoire- 3 to 5 Months" based on video recordings of infants with Prader-Willi syndrome. BMC Pediatr..

[22] Irshad M.T., Nisar M.A., Gouverneur P., Rapp M., Grzegorzek M. (2020). AI approaches towards Prechtl’s assessment of general movements: A systematic literature review. Sensors.

[23] Reich S., Zhang D., Kulvicius T., Bölte S., Nielsen-Saines K., Pokorny F.B., Peharz R., Poustka L., Wörgötter F., Einspieler C. (2021). Novel AI driven approach to classify infant motor functions. Sci. Rep..

[24] Groos D., Adde L., Aubert S., Boswell L., de Regnier R.A., Fjørtoft T., Gaebler-Spira D., Haukeland A., Loennecken M., Msall M. (2022). Development and validation of a deep learning method to predict cerebral palsy from spontaneous movements in infants at high risk. JAMA Netw. Open.

[25] Marschik P.B., Kulvicius T., Flügge S., Widmann C., Nielsen-Saines K., Schulte-Rüther M., Hüning B., Bölte S., Poustka L., Sigafoos J. (2022). Open video data sharing in developmental and behavioural science. arXiv.

[26] Kulvicius T., Zhang D., Nielsen-Saines K., Bölte S., Kraft M., Einspieler C., Poustka L., Wörgötter F., Marschik P.B. (2022). Infant movement classification through pressure distribution analysis-added value for research and clinical implementation. arXiv.

[27] Crowle C., Jackman M., Morgan C. (2023). The general movements motor optimality score in high-risk infants: A systematic scoping review. Pediatr. Phys. Ther..

[28] Butler M.G., Miller J.L., Forster J.L. (2019). Prader-Willi syndrome-clinical genetics, diagnosis and treatment approaches: An update. Curr. Pediatr. Rev..

[29] Tomantschger I., Herrero D., Einspieler C., Hamamura C., Voos M.C., Marschik P.B. (2018). The general movement assessment in non-European low- and middle-income countries. Rev. Saude Publica.

[30] Kaler J., Hussain A., Patel S., Majhi S. (2020). Neuromuscular junction disorders and floppy infant syndrome: A comprehensive review. Cureus.

[31] Sparks S.E. (2015). Neonatal hypotonia. Clin. Perinatol..

[32] Marschik P.B., Pokorny F.B., Peharz R., Zhang D., O’Muircheartaigh J., Roeyers H., Bölte S., Spittle A.J., Urlesberger B., Schuller B. (2017). A novel way to measure and predict development: A heuristic approach to facilitate the early detection of neurodevelopmental disorders. Curr. Neurol. Neurosci. Rep..

[33] Einspieler C., Prayer D., Marschik P.B. (2021). Fetal movements: The origin of human behaviour. Dev. Med. Child Neurol..

[34] Lord C., Brugha T.S., Charman T., Cusack J., Dumas G., Frazier T., Jones E.J.H., Jones R.M., Pickles A., State M.W. (2020). Autism spectrum disorder. Nat. Rev. Dis. Primers.

