# Supplementary material for: Building Blocks for Deep Phenotyping in Infancy: A Use Case Comparing Spontaneous Neuromotor Functions in Prader-Willi Syndrome and Cerebral Palsy
Source: J Clin Med. 2023 Jan 18;12(3):784. doi: 10.3390/jcm12030784 (PMC9917638; doi:10.3390/jcm12030784)
Supplement: Supplementary file 1 [file jcm-12-00784-s001.zip › jcm-2137657-supplementary.pdf]

Supplementary Materials:

Table S1. Perinatal risk factors in infants with Prader-Willi syndrome (PWS), those with cerebral palsy (CP), and infants with inconspicuous developmental outcomes (IOs). Each group had 18 cases. Data were extracted from the anamnesis records of the infants at the Rehabilitation Department of Children's Hospital at Fudan University, Shanghai, China. The numbers represent observed frequencies in each group.

Risk factor	PWS	CP	IOs
**Birth and maternal data**			
Birth weight (g)			
1500g< BW<2500g	4	4	3
1000g< BW<1500g	0	0	1
BW<1000g	0	0	0
Premature rupture of membranes	1	1	1
Antenatal steroids	0	0	1
Fetal intrauterine distress	3	3	0
Feeding difficulty	17	0	0
Weak cry	18	0	0
Hypotonia	17	0	0
Birth asphyxia	4	5	3
Apgar score<7	0	2	2
**Interval complications**			
(*From birth to term MRI*)			
Patent ductus arteriosus	3	3	2
Any intraventricular hemorrhage	2	3	3
Periventricular leukomalacia	0	7	0
Hypoxic-ischemic encephalopathy	0	4	0
NEC diagnosed or suspected	0	1	0
Confirmed sepsis	1	2	3
Bronchopulmonary dysplasia	0	1	0
Hyperbilirubinemia	3	10	6
**Abnormal MRI findings**			
Abnormal white matter signal	0	0	2
Encephalomalacia	0	12	1
Other abnormalities [a]	4	4	1

[a] including: widening of extracerebral space, delayed myelination, thinner corpus callosum, full lateral ventricles, widening of ventricles, reduced white matter
